# (*E*)-*N*′-[(2-Hydroxynaphthalen-1-yl)methylidene]nicotinohydrazide

**DOI:** 10.1107/S1600536811023257

**Published:** 2011-06-22

**Authors:** Shi-Yong Liu, Qin-Qin Guo, Yu-Mei Hao, Xiao-Ling Wang

**Affiliations:** a College of Chemistry and Pharmacy, Taizhou University, Taizhou Zhejiang 317000, People’s Republic of China; b Department of Chemistry, Baicheng Normal University, Baicheng 137000, People’s Republic of China; c Department of Chemistry, Liaoning Normal University, Dalian 116029, People’s Republic of China

## Abstract

In the mol­ecule of the title compound, C_17_H_13_N_3_O_2_, the naphthyl ring system and the pyridine ring form a dihedral angle of 12.2 (3)°. An intra­molecular O—H⋯N hydrogen bond generates a six-membered ring with an *S*(6) ring motif. This also contributes to the relative overall near planarity of the mol­ecule [r.m.s. deviation of all 22 non-H atoms = 0.107 (5) Å]. In the crystal, mol­ecules are linked through inter­molecular N—H⋯N hydrogen bonds, forming chains along the *a* axis.

## Related literature

For the medicinal applications of hydrazone compounds, see: Hillmer *et al.* (2010 ▶); Zhu *et al.* (2009 ▶). For hydrazones we have reported previously, see: Liu & You (2010 ▶); Liu & Wang (2010 ▶). For related structures, see: Khaledi *et al.* (2009 ▶); Xu *et al.* (2009 ▶); Shafiq *et al.* (2009 ▶). For hydrogen-bond motifs, see: Bernstein *et al.* (1995 ▶).
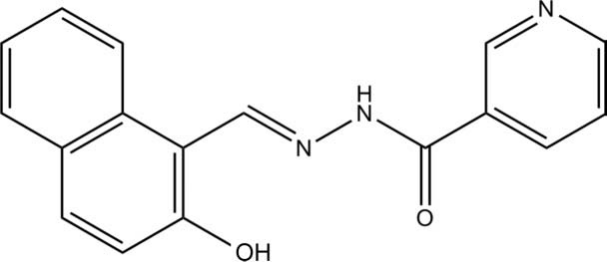


## Experimental

### 

#### Crystal data

C_17_H_13_N_3_O_2_
*M* *_r_* = 291.30
Orthorhombic, 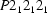

*a* = 6.253 (2) Å
*b* = 12.335 (4) Å
*c* = 18.511 (7) Å
*V* = 1427.8 (9) Å^3^
*Z* = 4
Mo *K*α radiation
μ = 0.09 mm^−1^
*T* = 298 K
0.20 × 0.20 × 0.18 mm

#### Data collection

Bruker SMART CCD area-detector diffractometer
Absorption correction: multi-scan (*SADABS*; Bruker, 2001 ▶) *T*_min_ = 0.982, *T*_max_ = 0.984
8787 measured reflections
1810 independent reflections
921 reflections with *I* > 2σ(*I*)
*R*_int_ = 0.081

#### Refinement

*R*[*F* ^2^ > 2σ(*F* ^2^)] = 0.049
*wR*(*F* ^2^) = 0.131
*S* = 0.99
1810 reflections
204 parameters
1 restraint
H atoms treated by a mixture of independent and constrained refinement
Δρ_max_ = 0.13 e Å^−3^
Δρ_min_ = −0.13 e Å^−3^

### 

Data collection: *SMART* (Bruker, 2007 ▶); cell refinement: *SAINT* (Bruker, 2007 ▶); data reduction: *SAINT*; program(s) used to solve structure: *SHELXTL* (Sheldrick, 2008 ▶); program(s) used to refine structure: *SHELXTL*; molecular graphics: *SHELXTL*; software used to prepare material for publication: *SHELXTL*.

## Supplementary Material

Crystal structure: contains datablock(s) global, I. DOI: 10.1107/S1600536811023257/sj5164sup1.cif

Structure factors: contains datablock(s) I. DOI: 10.1107/S1600536811023257/sj5164Isup2.hkl

Supplementary material file. DOI: 10.1107/S1600536811023257/sj5164Isup3.cml

Additional supplementary materials:  crystallographic information; 3D view; checkCIF report

## Figures and Tables

**Table 1:** Hydrogen-bond geometry (Å, °)

*D*—H⋯*A*	*D*—H	H⋯*A*	*D*⋯*A*	*D*—H⋯*A*
O1—H1⋯N1	0.82	1.85	2.565 (4)	146
N2—H2⋯N3^i^	0.90 (1)	2.20 (2)	3.066 (5)	161 (4)

Symmetry code: (i) 
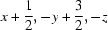
.

**Fig. 1.**
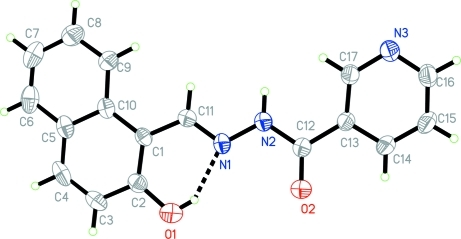
The molecular structure of the title compound. Displacement ellipsoids are drawn at the 30% probability level. Hydrogen atoms are shown as spheres of arbitrary radius and the intramolecular hydrogen bond is drawn as a dashed line.

**Fig. 2.**
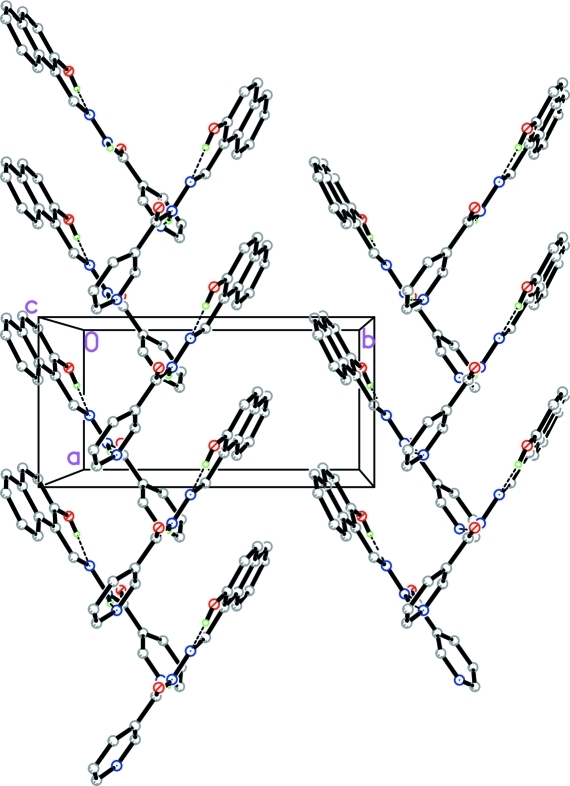
The molecular packing of the title compound, viewed along the c axis. Hydrogen bonds are shown as dashed lines. Hydrogen atoms not involved in hydrogen bonding have been omitted.

## supplementary crystallographic information

### Comment

Considerable attention has been focused on hydrazones and their medicinal applications (Hillmer *et al.*, 2010; Zhu *et al.*, 2009). A study of the crystal structures of such compounds is of particular interest (Khaledi *et al.*, 2009). As a continuation of our work on the preparation and structure of such compounds (Liu & You, 2010; Liu & Wang, 2010), we report herein the crystal structure of the title compound, a new hydrazone.

The molecular structure of the title compound is shown in Fig. 1. The dihedral angle between the C1—C10 naphthyl ring and the C13—C17/N3 pyridine ring is 12.2 (3) °. An intramolecular O1—H1···N1 hydrogen bond forms a six-membered ring, with an *S*(6) ring motif [Fig. 1] and contributes to the overall planarity of the molecule (Bernstein *et al.*, 1995). All the bond lengths are comparable to those observed in related structures (Xu *et al.*, 2009; Shafiq *et al.*, 2009) and those we reported previously. In the crystal structure, molecules are linked through intermolecular N2–H2···N3 hydrogen bonds (Table 1), to form chains along the *a* axis (Fig. 2).

### Experimental

The title compound was prepared by the condensation reaction of 2-hydroxy-1-naphthaldehyde (1.0 mmol, 0.172 g) and nicotinohydrazide (1.0 mmol, 0.137 g) in methanol (50 ml) at ambient temperature. Colourless block-shaped single crystals suitable for X-ray structural determination were obtained by slow evaporation of the solution for a few days.

### Refinement

H2 was located from a difference Fourier map and refined isotropically, with the N–H distance restrained to 0.90 (1) Å. The remaining H atoms were positioned geometrically and constrained to ride on their parent atoms, with C–H distances of 0.93 Å, O–H distance of 0.82 Å, and with *U*_iso_(H) = 1.2*U*_eq_(C) and 1.5*U*_eq_(O). In the absence of significant anomalous dispersion effects, 1294 Friedel pairs were averaged.

### Crystal data

**Table:**

C_17_H_13_N_3_O_2_	*F*(000) = 608
*M**_r_* = 291.30	*D*_x_ = 1.355 Mg m^−^^3^
Orthorhombic, *P*2_1_2_1_2_1_	Mo *K*α radiation, λ = 0.71073 Å
Hall symbol: P 2ac 2ab	Cell parameters from 536 reflections
*a* = 6.253 (2) Å	θ = 2.6–24.5°
*b* = 12.335 (4) Å	µ = 0.09 mm^−^^1^
*c* = 18.511 (7) Å	*T* = 298 K
*V* = 1427.8 (9) Å^3^	Block, colourless
*Z* = 4	0.20 × 0.20 × 0.18 mm

### Data collection

**Table:**

Bruker SMART CCD area-detector diffractometer	1810 independent reflections
Radiation source: fine-focus sealed tube	921 reflections with *I* > 2σ(*I*)
graphite	*R*_int_ = 0.081
ω scans	θ_max_ = 27.0°, θ_min_ = 2.0°
Absorption correction: multi-scan (*SADABS*; Bruker, 2001)	*h* = −7→7
*T*_min_ = 0.982, *T*_max_ = 0.984	*k* = −15→14
8787 measured reflections	*l* = −23→20

### Refinement

**Table:**

Refinement on *F*^2^	Secondary atom site location: difference Fourier map
Least-squares matrix: full	Hydrogen site location: inferred from neighbouring sites
*R*[*F*^2^ > 2σ(*F*^2^)] = 0.049	H atoms treated by a mixture of independent and constrained refinement
*wR*(*F*^2^) = 0.131	*w* = 1/[σ^2^(*F*_o_^2^) + (0.0547*P*)^2^] where *P* = (*F*_o_^2^ + 2*F*_c_^2^)/3
*S* = 0.99	(Δ/σ)_max_ = 0.001
1810 reflections	Δρ_max_ = 0.13 e Å^−^^3^
204 parameters	Δρ_min_ = −0.13 e Å^−^^3^
1 restraint	Extinction correction: *SHELXTL* (Sheldrick, 2008), Fc^*^=kFc[1+0.001xFc^2^λ^3^/sin(2θ)]^-1/4^
Primary atom site location: structure-invariant direct methods	Extinction coefficient: 0.020 (3)

### Special details

**Table:**

Geometry. All esds (except the esd in the dihedral angle between two l.s. planes) are estimated using the full covariance matrix. The cell esds are taken into account individually in the estimation of esds in distances, angles and torsion angles; correlations between esds in cell parameters are only used when they are defined by crystal symmetry. An approximate (isotropic) treatment of cell esds is used for estimating esds involving l.s. planes.
Refinement. Refinement of F^2^ against ALL reflections. The weighted R-factor wR and goodness of fit S are based on F^2^, conventional R-factors R are based on F, with F set to zero for negative F^2^. The threshold expression of F^2^ > 2sigma(F^2^) is used only for calculating R-factors(gt) etc. and is not relevant to the choice of reflections for refinement. R-factors based on F^2^ are statistically about twice as large as those based on F, and R- factors based on ALL data will be even larger.

### Fractional atomic coordinates and isotropic or equivalent isotropic displacement parameters (Å^2^)

**Table:**

	*x*	*y*	*z*	*U*_iso_*/*U*_eq_	
O1	0.7202 (5)	0.4933 (3)	0.16836 (14)	0.0776 (10)	
H1	0.6042	0.5236	0.1638	0.116*	
O2	0.2100 (5)	0.6667 (3)	0.21269 (15)	0.0921 (11)	
N1	0.4016 (5)	0.5735 (3)	0.09932 (17)	0.0550 (9)	
N2	0.2169 (6)	0.6340 (3)	0.09272 (17)	0.0556 (9)	
N3	−0.3466 (6)	0.8162 (3)	0.06814 (18)	0.0623 (10)	
C1	0.6656 (6)	0.4675 (3)	0.0409 (2)	0.0458 (9)	
C2	0.7836 (7)	0.4540 (3)	0.1040 (2)	0.0556 (11)	
C3	0.9784 (7)	0.3984 (3)	0.1031 (3)	0.0682 (13)	
H3	1.0544	0.3890	0.1459	0.082*	
C4	1.0576 (7)	0.3579 (3)	0.0404 (3)	0.0699 (13)	
H4	1.1884	0.3220	0.0410	0.084*	
C5	0.9465 (7)	0.3688 (3)	−0.0257 (3)	0.0577 (11)	
C6	1.0301 (8)	0.3281 (3)	−0.0913 (3)	0.0772 (14)	
H6	1.1625	0.2938	−0.0914	0.093*	
C7	0.9201 (10)	0.3384 (4)	−0.1541 (3)	0.0875 (16)	
H7	0.9771	0.3110	−0.1968	0.105*	
C8	0.7219 (9)	0.3898 (4)	−0.1546 (2)	0.0768 (14)	
H8	0.6467	0.3963	−0.1978	0.092*	
C9	0.6364 (7)	0.4310 (3)	−0.0923 (2)	0.0609 (11)	
H9	0.5034	0.4646	−0.0937	0.073*	
C10	0.7465 (7)	0.4234 (3)	−0.0255 (2)	0.0501 (10)	
C11	0.4700 (6)	0.5284 (3)	0.0414 (2)	0.0498 (10)	
H11	0.3917	0.5354	−0.0011	0.060*	
C12	0.1344 (7)	0.6808 (3)	0.1530 (2)	0.0592 (11)	
C13	−0.0594 (7)	0.7491 (3)	0.1416 (2)	0.0532 (11)	
C14	−0.1289 (10)	0.8127 (4)	0.1977 (2)	0.0980 (19)	
H14	−0.0557	0.8128	0.2414	0.118*	
C15	−0.3062 (10)	0.8761 (5)	0.1888 (3)	0.116 (2)	
H15	−0.3548	0.9196	0.2264	0.140*	
C16	−0.4116 (8)	0.8747 (4)	0.1241 (2)	0.0704 (13)	
H16	−0.5343	0.9167	0.1191	0.085*	
C17	−0.1735 (7)	0.7550 (3)	0.0787 (2)	0.0584 (11)	
H17	−0.1266	0.7129	0.0402	0.070*	
H2	0.165 (7)	0.648 (3)	0.0485 (11)	0.080*	

### Atomic displacement parameters (Å^2^)

**Table:**

	*U*^11^	*U*^22^	*U*^33^	*U*^12^	*U*^13^	*U*^23^
O1	0.085 (3)	0.085 (2)	0.0627 (19)	0.008 (2)	−0.0107 (17)	0.0032 (16)
O2	0.088 (3)	0.135 (3)	0.0539 (18)	0.046 (2)	−0.0086 (17)	−0.0062 (18)
N1	0.046 (2)	0.0574 (19)	0.062 (2)	0.0066 (18)	0.0041 (18)	−0.0025 (18)
N2	0.048 (2)	0.064 (2)	0.055 (2)	0.0080 (19)	0.0020 (18)	−0.0004 (18)
N3	0.055 (3)	0.064 (2)	0.068 (2)	0.004 (2)	0.0006 (19)	−0.0009 (18)
C1	0.041 (2)	0.041 (2)	0.056 (2)	−0.0007 (19)	0.0009 (19)	0.0080 (18)
C2	0.056 (3)	0.048 (2)	0.063 (3)	−0.002 (2)	−0.006 (2)	0.005 (2)
C3	0.060 (3)	0.063 (3)	0.081 (3)	0.006 (3)	−0.019 (3)	0.013 (3)
C4	0.047 (3)	0.045 (2)	0.117 (4)	0.007 (2)	−0.008 (3)	0.011 (3)
C5	0.048 (3)	0.038 (2)	0.087 (3)	−0.004 (2)	0.006 (3)	0.003 (2)
C6	0.054 (3)	0.061 (3)	0.117 (4)	−0.006 (3)	0.031 (3)	−0.014 (3)
C7	0.092 (4)	0.081 (3)	0.089 (4)	−0.007 (3)	0.028 (3)	−0.027 (3)
C8	0.089 (4)	0.078 (3)	0.064 (3)	−0.007 (3)	0.005 (3)	−0.001 (2)
C9	0.066 (3)	0.061 (2)	0.056 (2)	−0.001 (2)	0.004 (2)	−0.002 (2)
C10	0.045 (3)	0.041 (2)	0.064 (3)	−0.002 (2)	0.007 (2)	0.0061 (19)
C11	0.050 (3)	0.050 (2)	0.049 (2)	0.001 (2)	−0.0025 (19)	0.002 (2)
C12	0.056 (3)	0.070 (3)	0.052 (3)	0.010 (3)	0.004 (2)	−0.001 (2)
C13	0.058 (3)	0.054 (2)	0.047 (2)	0.008 (2)	0.004 (2)	0.0012 (19)
C14	0.122 (5)	0.121 (4)	0.051 (3)	0.066 (4)	−0.008 (3)	−0.018 (3)
C15	0.153 (6)	0.144 (5)	0.052 (3)	0.092 (5)	0.008 (3)	−0.010 (3)
C16	0.068 (3)	0.074 (3)	0.070 (3)	0.020 (3)	0.011 (3)	0.009 (3)
C17	0.052 (3)	0.063 (3)	0.061 (3)	0.003 (2)	0.001 (2)	−0.012 (2)

### Geometric parameters (Å, °)

**Table:**

O1—C2	1.346 (4)	C6—C7	1.357 (7)
O1—H1	0.8200	C6—H6	0.9300
O2—C12	1.214 (4)	C7—C8	1.393 (7)
N1—C11	1.282 (4)	C7—H7	0.9300
N1—N2	1.381 (4)	C8—C9	1.370 (6)
N2—C12	1.358 (5)	C8—H8	0.9300
N2—H2	0.898 (10)	C9—C10	1.417 (5)
N3—C16	1.326 (5)	C9—H9	0.9300
N3—C17	1.334 (5)	C11—H11	0.9300
C1—C2	1.392 (5)	C12—C13	1.491 (6)
C1—C11	1.435 (5)	C13—C17	1.367 (5)
C1—C10	1.436 (5)	C13—C14	1.372 (5)
C2—C3	1.398 (6)	C14—C15	1.366 (6)
C3—C4	1.357 (6)	C14—H14	0.9300
C3—H3	0.9300	C15—C16	1.368 (6)
C4—C5	1.414 (6)	C15—H15	0.9300
C4—H4	0.9300	C16—H16	0.9300
C5—C6	1.414 (6)	C17—H17	0.9300
C5—C10	1.421 (5)		
			
C2—O1—H1	109.5	C7—C8—H8	119.7
C11—N1—N2	116.1 (3)	C8—C9—C10	121.4 (4)
C12—N2—N1	118.3 (3)	C8—C9—H9	119.3
C12—N2—H2	122 (3)	C10—C9—H9	119.3
N1—N2—H2	119 (3)	C9—C10—C5	117.2 (4)
C16—N3—C17	116.2 (4)	C9—C10—C1	123.4 (4)
C2—C1—C11	120.6 (4)	C5—C10—C1	119.4 (4)
C2—C1—C10	119.1 (4)	N1—C11—C1	121.1 (4)
C11—C1—C10	120.2 (4)	N1—C11—H11	119.4
O1—C2—C1	122.9 (4)	C1—C11—H11	119.4
O1—C2—C3	116.3 (4)	O2—C12—N2	122.6 (4)
C1—C2—C3	120.7 (4)	O2—C12—C13	121.8 (4)
C4—C3—C2	120.5 (4)	N2—C12—C13	115.6 (4)
C4—C3—H3	119.7	C17—C13—C14	116.6 (4)
C2—C3—H3	119.7	C17—C13—C12	125.1 (4)
C3—C4—C5	121.8 (4)	C14—C13—C12	118.3 (4)
C3—C4—H4	119.1	C15—C14—C13	119.6 (5)
C5—C4—H4	119.1	C15—C14—H14	120.2
C6—C5—C4	121.9 (5)	C13—C14—H14	120.2
C6—C5—C10	119.7 (4)	C14—C15—C16	119.3 (5)
C4—C5—C10	118.4 (4)	C14—C15—H15	120.4
C7—C6—C5	121.0 (5)	C16—C15—H15	120.4
C7—C6—H6	119.5	N3—C16—C15	122.9 (5)
C5—C6—H6	119.5	N3—C16—H16	118.5
C6—C7—C8	120.0 (5)	C15—C16—H16	118.5
C6—C7—H7	120.0	N3—C17—C13	125.4 (4)
C8—C7—H7	120.0	N3—C17—H17	117.3
C9—C8—C7	120.7 (5)	C13—C17—H17	117.3
C9—C8—H8	119.7		

### Hydrogen-bond geometry (Å, °)

**Table:**

*D*—H···*A*	*D*—H	H···*A*	*D*···*A*	*D*—H···*A*
O1—H1···N1	0.82	1.85	2.565 (4)	146
N2—H2···N3^i^	0.90 (1)	2.20 (2)	3.066 (5)	161 (4)

Symmetry codes: (i) *x*+1/2, −*y*+3/2, −*z*.
